# Transient Liquid Phase Bonding of Hastelloy X with Inconel 738 Superalloy Using BNi-2 Interlayer: Microstructure and Mechanical Properties

**DOI:** 10.3390/ma19020227

**Published:** 2026-01-06

**Authors:** Lin Yang, Yuwei Zhao, Xingdong Chen, Ke Li, Xingyu Zhang, Panpan Lin, Tiesong Lin, Peng He

**Affiliations:** 1 State Key Laboratory of Clean and Efficient Turbomachinery Power Equipment, Deyang 618000, China; dtcyanglin@dongfang.com; 2 Dongfang Electric Corporation Dongfang Turbine Co., Ltd., Deyang 618000, China; 3 State Key Laboratory of Precision Welding & Joining of Materials and Structures, Harbin Institute of Technology, Harbin 150001, China; zywhitm@163.com (Y.Z.); 17803850689@163.com (K.L.); 24s109346@stu.hit.edu.cn (X.Z.); pplin@hit.edu.cn (P.L.); hitjoining@hit.edu.cn (T.L.); 4 Zhengzhou Research Institute, Harbin Institute of Technology, Zhengzhou 450000, China; 5 Zhengzhou Research Institute for Abrasives & Grinding Co., Ltd., Zhengzhou 450001, China

**Keywords:** boride precipitation, mechanical property, transient liquid phase bonding

## Abstract

The dissimilar joining of solid-solution-strengthened superalloys and precipitation-strengthened superalloys enables complementary performance synergy, holding significant application potential in the aerospace industry. This study investigated the transient liquid phase bonding of Hastelloy X and IN738 using a BNi-2 interlayer, focusing on the effects of bonding temperature and time on interfacial microstructure evolution and mechanical properties. The results demonstrated that achieving complete isothermal solidification is paramount for joint properties, a process governed by the synergistic control of bonding temperature and time. At lower temperatures (e.g., 1050 °C), the joint centerline contained an athermal solidification zone (ASZ) rich in hard and brittle Cr-rich (∼15.9 GPa) and Ni-rich borides, which served as the failure initiation site. As the ASZ was progressively eliminated with increasing temperature, a fully isothermal solidified zone (ISZ, ∼52 μm wide) consisting of γ-Ni formed at 1100 °C. Concurrently, Cr-Mo borides (∼9.8 GPa) precipitated within the diffusion-affected zone (DAZ) on the Hastelloy X side, becoming the new potential sites for crack initiation. Prolonging the holding time at 1100 °C not only ensured complete isothermal solidification but also promoted Mo diffusion, which improved the plasticity of the Cr-Mo borides and their interfacial bonding with the γ-Ni matrix (∼5.9 GPa). This synergistic optimization resulted in a significant increase in joint shear strength, achieving a maximum value of 587 MPa under the optimal condition of 1100 °C/40 min.

## 1. Introduction

The gas turbine serves as a core energy conversion device in the aerospace and energy sectors. With the continuous improvement of engine performance, turbine inlet temperatures have been steadily rising, placing increasingly stringent demands on hot-end components [1,2,3]. The complementary properties of IN738 [4]—notably its exceptional high-temperature strength and creep resistance—and Hastelloy X [5], recognized for its superior oxidation and corrosion resistance at elevated temperatures, present a significant opportunity for advanced material systems, particularly in applications requiring both high strength and environmental durability. The effective integration of these alloys through tailored material design and manufacturing processes holds the potential to enable significant advances in the fabrication of complex high-temperature components used in turbine engines, with considerable promise for future applications [6,7,8]. Consequently, achieving high-integrity bonding of these two dissimilar superalloys has become a critical technological challenge in the manufacturing process.

However, traditional joining techniques like fusion welding and conventional brazing face difficulties here. Fusion welding joints are prone to solidification cracking, which is often accompanied by compositional segregation [9,10,11], while conventional brazing processes often form continuous brittle intermetallic compounds in the joints [12], which severely degrade the high-temperature mechanical properties of the joint. Transient Liquid Phase (TLP) bonding technology offers a promising solution to this challenge. This technique employs an interlayer containing melting point depressant (MPD) elements (e.g., B, Si) [13]. At the bonding temperature, the interlayer melts, forming a liquid phase that wets and fills the joint gap. Subsequently, the out-diffusion of MPD elements into the base metals enables isothermal solidification, ultimately producing a homogeneous joint with a composition and properties close to those of the base metal [14,15,16]. The key to obtaining a high-performance joint lies in achieving complete isothermal solidification [17,18,19].

The TLP process typically consists of four stages: heating/melting, base metal dissolution, isothermal solidification, and homogenization [8]. Among these, isothermal solidification is the core characteristic stage. Its completion directly determines whether low-melting-point eutectic phases remain in the joint centerline, thereby influencing the joint’s high-temperature strength and ductility [20]. Bonding temperature and bonding time are the two critical process parameters controlling the kinetics of isothermal solidification. An excessively low temperature significantly reduces the diffusion rate of the MPD elements. Conversely, an excessively high temperature leads to excessive dissolution of the base metal, increasing the width of the liquid zone and the diffusion distance for MPD elements, which can retard or even prevent the completion of isothermal solidification [21]. Similarly, the bonding time must be precisely controlled: an insufficient duration prevents adequate diffusion of MPD elements [22], resulting in incomplete isothermal solidification and the formation of brittle eutectic constituents at the joint centerline; conversely, an excessively long duration may lead to grain coarsening in the joint region, which is also detrimental to mechanical properties. Therefore, systematic investigation into the synergistic effects of bonding temperature and time on the microstructural evolution of the dissimilar joint is crucial.

Although TLP bonding has been widely applied to various nickel-based superalloys, systematic studies on the combination of Hastelloy X (strengthened by solid solution) and IN738 (strengthened by precipitation), using a BNi-2 filler alloy, remain scarce. In particular, three key aspects are yet to be fully elucidated for this specific dissimilar joint: (i) the systematic correlation between process parameters and the evolution of interfacial zones (ASZ/ISZ/DAZ); (ii) the quantitative relationship between the composition/plasticity of precipitates (especially Cr–Mo borides) and the macroscopic mechanical properties; and (iii) the mechanistic origin of asymmetric boride precipitation within the ASZ, linked to the distinct base metal microstructures. Based on this, the present work employs a BNi-2 interlayer to TLP bond Hastelloy X and IN738. It focuses on investigating the influence of bonding temperature and time on the interfacial microstructure and the evolution of precipitates. Combined with nanoindentation and shear testing, the fracture mechanism of the joints is thoroughly analyzed. This study aims to provide a novel and comprehensive understanding of the microstructure-property relationships in this dissimilar joint system, offering a reliable theoretical basis and practical guidance for achieving high-quality bonding.

## 2. Materials and Methods

### 2.1. Materials and TLP Bonding Process

In this work, IN738 and Hastelloy X were used as the base materials for joining. In addition, the filler used for bonding was BNi-2. The solidus and liquidus temperatures of the BNi-2 filler were 971 °C and 999 °C, respectively. The nominal chemical compositions of the base and filler metals are given in Table 1. The IN738 alloy contained the γ-Ni solid solution, the strengthening precipitates γ’-Ni_3_ (Al, Ti) and dispersed carbides, and the Hastelloy X primarily consisted of γ-Ni matrix with solid solution strengthening elements (such as Mo, Cr, W) that provide the main strengthening effect and dispersed carbides.

**Figure 1:**
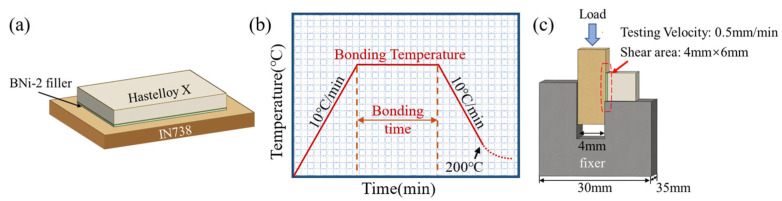
Schematic diagrams of (**a**) The assembly of Hastelloy X/IN738 joint; (**b**) the bonding thermal cycle of the joint; (**c**) shear test.

The base metals were sectioned into specimens with dimensions of 6 mm × 8 mm for IN738 and 4 mm × 6 mm for Hastelloy X using wire electrical discharge machining (EDM). All specimen surfaces were progressively ground with SiC emery papers from 80-grit to 1500-grit. Subsequently, they were ultrasonically cleaned in acetone for 10 min and dried. The Hastelloy X specimen was coated with the filler paste by using a screen printing method with a thickness of 100 µm. And the Hastelloy X specimen was then assembled onto the IN738 specimen, as illustrated in Figure 1a. Graphite blocks were placed on top of the assembly to secure the specimens. Finally, the assembled specimens were subjected to TLP bonding in a vacuum furnace(CENTORR, Nashua, NH, USA) (10^−5^ Torr) at 1050–1175 °C for 10–40 min. The bonding thermal cycle is presented in Figure 1b.

### 2.2. Microstructural and Mechanical Characterization

Following the bonding process, the cross-sections of the joints were ground with emery papers and subsequently polished with 50 nm SiO_2_ solution. The polished specimens were then examined using a scanning electron microscope (SEM) (Zeiss, Oberkochen, Germany) operated at 20 kV to observe the microstructure of the Hastelloy X/IN738 dissimilar joint, equipped with an energy dispersive spectroscopy (EDS) system. Elemental distribution were obtained by electron probe microanalyzer (EPMA) (JEOL, Tokyo, Japan). The chemical composition within selected regions was determined by wavelength-dispersive spectroscopy (WDS). Phase identification of the joint was performed using X-ray diffraction (XRD) (Panalytical, Almelo, The Netherlands) with Cu Kα radiation. The XRD patterns were acquired in a 2θ range from 10° to 90° with a step size of 0.04° and a scanning speed of 0.2 s/step. Nano-indentation tests were conducted at room temperature (RT) using a G200 nano-indenter (Aglient, Santa Clara, CA, USA) to evaluate the micro-hardness of the phases in the Hastelloy X/IN738 dissimilar joint. A total of 30 indents were made across the entire weld region of the joint under a maximum load of 10 mN with a dwell time of 10 s. The indents were arranged along a linear scan path oriented at 45° to the weld seam, spanning from one base metal through the weld to the other base metal, with a consistent spacing of 10 µm between adjacent measurement points. Room-temperature (RT) shear strength were tested with a universal testing machine (Shimadzu, Kyoto, Japan) at a constant speed of 0.5 mm/min, employing a shearing fixture as shown in Figure 1c. To minimize experimental error, three shear test specimens for each parameter were tested, and the average value was recorded.

## 3. Results and Discussion

### 3.1. Typical Microstructure of the TLP-Bonded Hastelloy X/IN738 Joint

**Figure 2:**
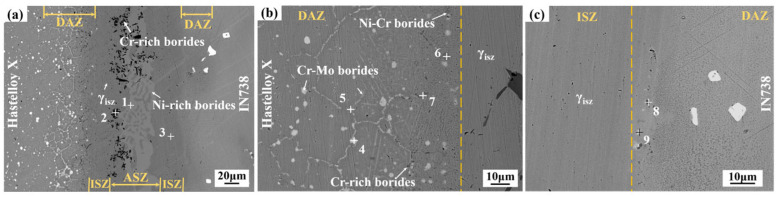
Microstructure of Hastelloy X/IN738 joint brazed at 1050 °C/30 min: (**a**) entire joint; (**b**) details of DAZ on Hastelloy X side; (**c**) details of DAZ on IN738 side.

Figure 2 presents the typical interfacial microstructure of the Hastelloy X/IN738 joint bonded at 1050 °C for 30 min. As seen in Figure 2a, the joint can be clearly divided into four distinct zones: the athermal solidification zone (ASZ) at the center, the isothermal solidification zone (ISZ) adjacent to it, and the diffusion affected zones (DAZ) on both the Hastelloy X and IN738 sides. Detailed morphologies of these zones are shown in Figure 2b,c. The ISZ primarily consists of a gray γ matrix (Phase 3). The ASZ is composed of irregularly dispersed black blocky/rod-like precipitates (Phase 2) and continuous light gray fishbone-like phase (Phase 1) with gray matrix incorporated in it. The morphology and distribution of phase in the DAZ differ significantly between the two sides. On the Hastelloy X side (see Figure 2b), white precipitates (Phase 5) along grain boundaries and granular gray precipitates (Phases 6, 7) are observed, with the latter located closer to the joint interface. In contrast, the DAZ on the IN738 side contains only a limited number of fine, blocky dark gray phase (Phase 8) and white granular precipitates.

**Figure 3:**
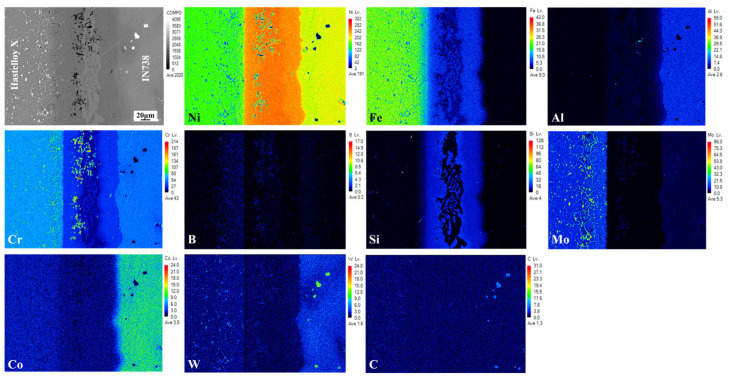
EPMA mapping results of Hastelloy X/IN738 joint brazed at 1050 °C/30 min.

The EPMA results of the joint are displayed in Figure 3, and the chemical compositions of the various phases in Figure 2 are listed in Table 2 (tested by EDS). Combined with the EDS results from Table 2, Phase 3 in the ISZ, and in the ASZ the gray matrix incorporated in fishbone-like phase are identified as Ni-based solid solution. The continuous light gray phases (phase 1) in the ASZ, rich in Ni and B elements, were determined to be Ni-rich borides. The black precipitates (phase 2), enriched in Cr and B, is identified as a Cr-rich boride. In the DAZ on the Hastelloy X side, phases marked as 4 and 5 are both Cr-Mo borides. Based on the elemental distribution, the discrete white phases (marked as 9) in DAZ (IN738 side) are Cr-Mo borides, while the granular phases (phase 8) are Cr-rich borides.

The formation of these microstructural features is attributed to the TLP boding process. The ISZ, primarily comprising γ solid solution, formed through isothermal solidification. Isothermal solidification initiated when the concentration of MPD atoms in the liquid decreased under their equilibrium solubility in the solid at the bonding temperature (denoted as C_γL_). This decrease in solute concentration triggers the precipitation of the γ_ISZ_ phase, a Ni-rich austenitic solid solution containing elements such as Cr, Fe, and W. However, under the condition of 1050 °C for 30 min, the limited diffusion of MPD atoms prevented complete isothermal solidification across the entire joint seam. Consequently, a residual liquid remained along the centerline upon cooling. Upon cooling, this residual liquid solidifies to form the ASZ (Figure 2a). The solidification involved the formation of primary γ phase, followed by a eutectic reaction that produced Ni-rich and Cr-rich borides. This solidification sequence is consistent with prior reports on filler metals containing MPD elements [23,24,25].

Figure 2b,c presents detailed morphologies of the DAZ on both sides of the joint. Previous studies reported that the formation of the DAZ is closely related to elemental diffusion and precipitation behavior. During the isothermal solidification and subsequent cooling, MPD atoms (e.g., B, Si) from the filler metal diffuse into the base metals and gradually enrich in the DAZ. Owing to the relatively low bonding temperature and time, the diffusion kinetics of MPD atoms are kinetically constrained, leading to accumulation of these elements within the DAZ. When the concentrations of B and Si exceed their respective solid solubility limits in the base metal, precipitates are formed within the DAZ. Based on the Ni-B and Ni-Si binary phase diagrams [26], the solid solubility of B and Si in Ni matrix at approximately 1050 °C is about 0.3 at.% and 15 at.%, respectively [8,27]. Combined with the EPMA results of the joint (Figure 3) and the EDS data from Table 2, it is evident that Si is primarily concentrated within the γ solid solution phase in the joint. Its concentration in both the ASZ and DAZ remains below its solid solubility limit, which prevents silicide formation. As a result, the precipitates in the DAZ are predominantly borides, consistent with previous studies that reported Si remains in the solid solution form and does not precipitate as silicide [28,29].

**Figure 4:**
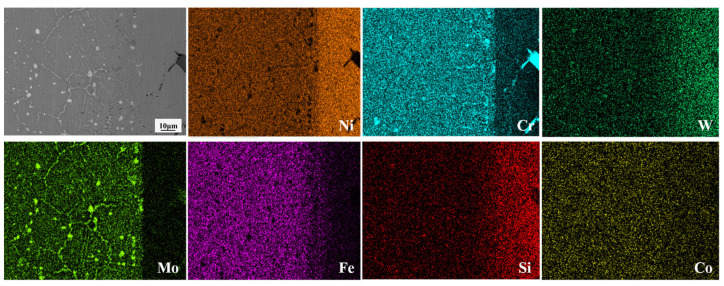
Microstructure and corresponding EDS maps of DAZ on the Hastelloy X side at 1050 °C/30 min.

Notable differences occurred in the composition and morphological distribution of these compounds between the DAZ (Hastelloy X side) and the DAZ (IN738 side) [30]. This asymmetry stems primarily from the distinct microstructural characteristics of the two base metals. Hastelloy X, a typical solid-solution strengthened superalloy, contains a high density of grain boundaries. The high grain boundary energy provides fast diffusion channels for B atoms. Once the solid solubility limit of B in the Ni-matrix was exceeded, the diffused B atoms reacted with the abundant boride-forming elements (e.g., Cr, Mo) in Hastelloy X, thereby inducing the nucleation and growth of boride precipitates. As evidenced by the elemental maps in Figure 4, two distinct boride morphologies are identified on the Hastelloy X side: (i) discrete, dark-contrast dot-shaped Cr-rich borides near the ISZ/DAZ interface, and (ii) bright-contrast Cr-Mo borides predominantly aligned along grain boundaries farther from the interface. This morphological distribution is attributed to the distinct diffusion kinetics and mechanisms of Cr, Mo, and B atoms. The fundamental precipitation mechanism will be elaborated in detail in following Section 3.5.

### 3.2. Joint Microstructure Evolution Versus Bonding Temperature

**Figure 5:**
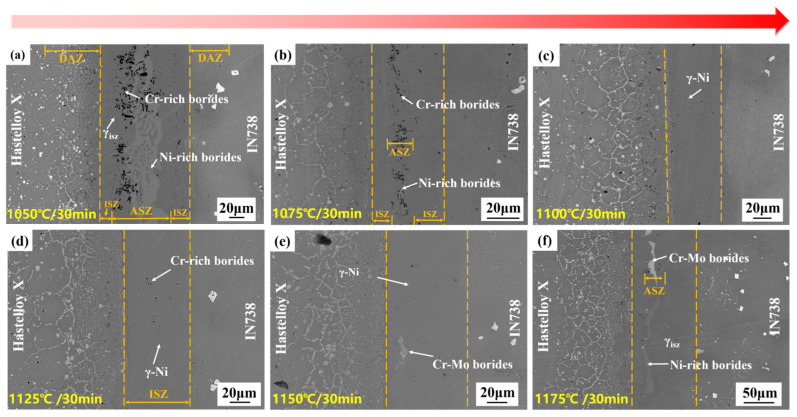
Microstructure of Hastelloy X/IN738 joints brazed at (**a**) 1050 °C for 30 min; (**b**) 1075 °C for 30 min; (**c**) 1100 °C for 30 min; (**d**) 1125 °C for 30 min; (**e**) 1150 °C for 30 min; (**f**) 1175 °C for 30 min.

The microstructure images of Hastelloy X/IN738 joints brazed at 1050–1175 °C for 30 min are illustrated in Figure 5. The study focused on elucidating the role of temperature in governing the isothermal solidification process and the governing of the precipitation and distribution of phases.

Under the condition of 1050 °C for 30 min, the insufficient diffusion behavior of MPD elements led to the formation of a joint predominated by ASZ. The ASZ contained numerous rod-like and blocky Cr-rich borides, accompanied by Ni-rich boride islands, all embedded in a γ-Ni matrix. In contrast, the ISZ exhibited a notably narrow width. With the increase in temperature, the diffusion coefficient of B atoms increased, the width of the ISZ expanded accordingly, and the number of borides decreased significantly. When the temperature rose to 1100 °C, complete isothermal solidification was achieved, forming a homogeneous ISZ with a width of 52 μm, exclusively consisting of γ-Ni. At elevated temperatures (1125–1175 °C), excessive dissolution of the base metal resulted in a significant increase in joint width; which further elongated the diffusion path, and in turn led to the retention of B atoms within the joint. Concurrently, the elevated thermal energy enabled Mo atoms to overcome the diffusion energy barrier, cooperating with Cr and B to form Cr-Mo borides. Notably, Ni-rich borides reappeared at 1175 °C, and this recurrence is attributed to excessive base metal dissolution (which widened the joint). The elongated diffusion path for B atoms, combined with the prior consumption of strong boride-forming elements (Cr and Mo) in the widened region, left residual B atoms to react with the more abundant but less favorable Ni atoms.

The bonding temperature serving as the pivotal parameter for isothermal solidification, fundamentally governs the process by synergistically controlling atomic diffusion kinetics and base metal dissolution thermodynamics.

On one hand, the increasing temperature lowers the diffusion activation energy of MPD elements (B, Si), thereby accelerating their migration into the base metals. This temperature dependence of the diffusion coefficient adheres to the Arrhenius equation [30]:

*D* = *D*_0_
*exp*(−*Q*/*RT*), (1)

In this equation, *D* represents the diffusion coefficient, *D*_0_ is the frequency factor; *Q* stands for the diffusion activation energy, *R* is the universal gas constant, and *T* is the absolute temperature. As derived from Equation (1), an increase in temperature significantly increases the value of the *exp*(−*Q*/*RT*) term, thereby enhancing the diffusion coefficient. This accelerated diffusion of B and Si atoms, in turn, promotes the isothermal solidification process.

On the other hand, excessive temperature elevation exacerbates base metal dissolution, thereby increasing the joint width (*W*). Li, et al. also observed a thickening of the isothermal solidification zone with increasing bonding temperature [31]. This elongation of the diffusion path for B atoms reduces their concentration gradient (∇*C*). According to Fick‘s first law, the diffusion flux (*J*), which governs the kinetics of isothermal solidification, is expressed as:

*J* = −*D* ∇*C*, (2)

where *D* is the diffusion coefficient of B (governed by Equation (1)). The concentration gradient (∇*C*) can be quantified by:

∇*C* = ∆*C*/*W*, (3)

where ∆*C* is the B concentration difference between the liquid at the joint centerline and the solid base metal. Consequently, an increase in *W* lowers ∇*C*, which in turn decreases the diffusion flux (*J*). This reduced flux ultimately hinders the completion of isothermal solidification.

In this study, 1100 °C was identified as the critical threshold for achieving complete isothermal solidification. At this temperature, an optimal balance is attained: the thermally enhanced diffusion coefficient (*D*) effectively counteracts the negative impact of the reduced concentration gradient (∇*C*) caused by moderate joint widening, while avoiding excessive base metal dissolution. Therefore, this temperature constitutes the optimal processing parameter.

### 3.3. Joint Microstructure Evolution Versus Bonding Time

**Figure 6:**
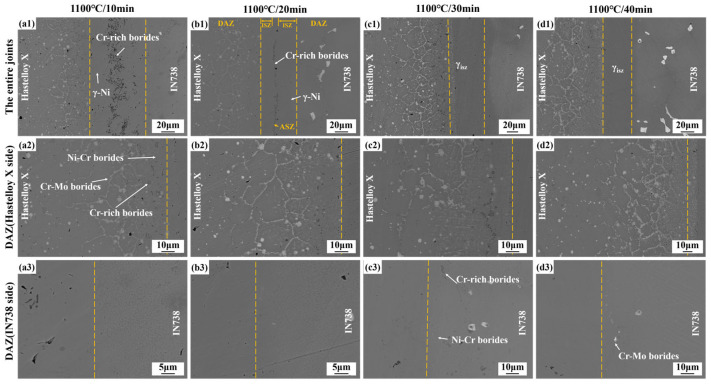
Microstructure of Hastelloy X/IN738 joints brazed at 1100 °C for (**a1**–**a3**) 10 min; (**b1**–**b3**) 20 min; (**c1**–**c3**) 30 min; (**d1**–**d3**) 40 min.

Figure 6 presents microstructural images of Hastelloy X/IN738 joints brazed at 1100 °C for 10–40 min. When brazed at 1100 °C for 10 min, incomplete isothermal solidification occurred, leaving a B-enriched residual liquid in ISZ. During the subsequent cooling stage, this liquid solidified under non-isothermal conditions, precipitating Cr-rich borides (dot-like and rod-like) along the joint centerline and formed ASZ. When the bonding time was extended to 20 min, a more sufficient diffusion duration for B atoms significantly reduced the content of Cr-rich borides, with the majority of the joint achieving isothermal solidification. As the bonding time was further increased to 30–40 min, the joint achieved complete isothermal solidification, fully composed of the γ-Ni matrix.

The detailed microstructural images of DAZ (Hastelloy X side) and DAZ (IN738 side) at 1100 °C for different time (10–40 min) can be seen in Figure 6. At a bonding time of 10 min, The DAZ (Hastelloy X side) exhibited gray dotted Ni-Cr borides, black dotted Cr-rich borides, and white Cr-Mo borides that precipitated primarily along grain boundaries. On the Hastelloy X side, prolonged brazing time enhanced elemental diffusion. This diffusion facilitated the dissolution of unstable Cr-rich borides. However, the incorporation of Mo stabilizes the boride phase in Cr-Mo borides, thereby inhibiting their dissolution. Concurrently, the DAZ (Hastelloy X side) width significantly increased.

In contrast, on the DAZ (IN738 side), precipitation of borides was markedly delayed and suppressed. Only amounts of Cr-rich borides and Ni-Cr borides were detected, when the bonding time reached 30 min. When brazed for 40 min, Cr-Mo borides underwent limited precipitation. This is located at the coherent γ precipitates not only impede boron diffusion but also prevent boride-forming elements in the substrate (such as Cr and W) from reacting with boron. These observations underscore that the DAZ evolution is governed by the synergistic effects of elemental diffusion and the thermodynamic stability of boride phases, which are intrinsically coupled to the base metal composition and microstructure.

These observations underscore that the DAZ evolution is governed by the synergistic effects of elemental diffusion and the thermodynamic stability of boride phases, which are intrinsically coupled to the base metal composition and microstructure. More importantly, the transition from fine, continuous Cr-rich borides to coarsened, discrete Cr-Mo borides with prolonged time signifies a microstructural optimization. This evolution not only reduces the continuity of hard and brittle networks but also, through the incorporation and sufficient diffusion of Mo, enhances the deformability and interfacial cohesion of the boride phases. These factors collectively dictate the mechanical response and fracture path of the joint, as will be discussed in Section 3.4.

### 3.4. Mechanical Properties of the Hastelloy X/IN738 Joints

**Figure 7:**
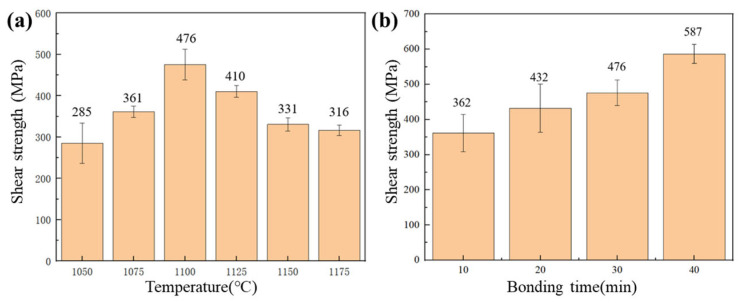
Shear test results of the Hastelloy X/IN738 joints brazed at (**a**) 1050–1175 °C for 30 min; (**b**) 1100 °C for different time (10–40 min).

The shear strength of the joints, as summarized in Figure 7, was significantly influenced by the brazing parameters. It exhibited a non-monotonic relationship with temperature, first increasing and then decreasing, while showing a consistent improvement with prolonged bonding time. The minimum strength of 285 MPa was obtained at 1050 °C/30 min, primarily due to the presence of ASZ rich in continuous brittle phases. As the temperature increased to 1100 °C, the ASZ was eliminated and complete isothermal solidification was achieved, leading to a 67% increase in strength to 476 MPa. A further temperature rise resulted in a performance degradation, as excessive base metal dissolution at elevated temperatures introduced fresh boride-forming elements into the joint, leading to the reprecipitation of brittle borides. Bonding time also played a critical role. At 1100 °C, a short bonding time of 10 min inhibited the sufficient diffusion of B within the joint, resulting in the precipitation of brittle Cr-rich borides in the ASZ (center of the joint) and a shear strength of 362 MPa. When the bonding time was extended to 30 min at 1100 °C, complete isothermal solidification was achieved, which eliminated the central brittle zone and resulted in a strength of 476 MPa. The further extension to 40 min yielded the peak shear strength of 587 MPa. This additional enhancement is attributed not only to the complete isothermal solidification but also, more importantly, to an advanced microstructural optimization within the DAZ on the Hastelloy X side. As described in Section 3.3, the prolonged bonding time promoted sufficient diffusion of Mo, leading to the coarsening and spheroidization of the Cr-Mo borides and significantly improving their interfacial bonding with the γ-Ni matrix. This microstructural evolution enhanced the toughness of the DAZ, which became the dominant fracture path, and allowed for greater plastic deformation energy dissipation during shear testing. The high strength is thus attributed to sufficient elemental diffusion and enhanced microstructural homogenization, which has been widely reported in the literature [31,32,33].

**Figure 8:**
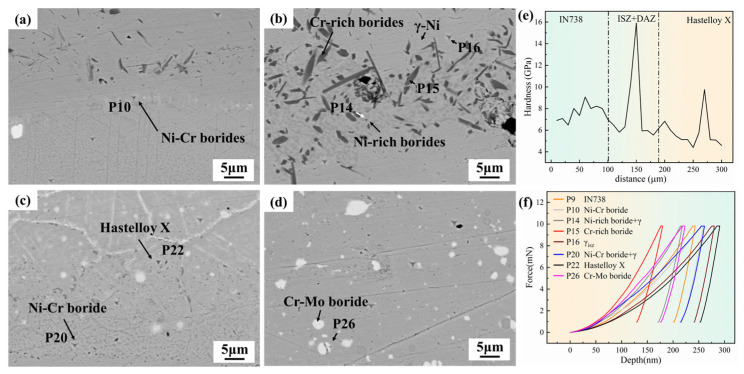
Nano-indentation tests on the Hastelloy X/IN738 joint brazed at 1050 °C/30 min: (**a**) SEM image of the DAZ (IN738 side); (**b**) SEM image of the ASZ; (**c**) SEM image of the DAZ (Hastelloy X side); (**d**) SEM image of the base metal(Hastelloy X); (**e**) distribution of the nano-hardness; (**f**) typical load–displacement curves of different regions.

Figure 8 shows the nano-indentation tests on the Hastelloy X/IN738 joint brazed at 1050 °C/30 min. Figure 8a,d present the SEM morphologies of indentations in different regions of the joint. It can be intuitively observed that the indentation pit sizes of Cr-rich boride (P15) and Ni-rich boride (P14) are significantly smaller than that of the γ-Ni (P16) (as shown in Figure 8b). This phenomenon reflects that the borides in the ASZ are much harder than the γ-Ni matrix phase in the seam. Hardness tests indicate that the hardness of Cr-rich borides (P15) is 15.919 GPa; while Ni-rich borides, due to their small size, precipitate in eutectic form with the γ-Ni phase, the tested hardness at point P14 (Ni-rich borides + γ-Ni) is 10.301 GPa—even with the inclusion of the matrix phase, this hardness is still much higher than the 5.935 GPa of the pure γ-Ni phase (P16). Figure 8e,f show the nanohardness distribution curve of the entire joint and typical load–displacement curves, respectively, further clarifying the spatial distribution characteristics of hard and brittle phases: in the ASZ region, hard and brittle phases Cr-rich borides (P15) and Ni-rich borides (P14) precipitated, which are the main weak zone of the joint; in the DAZ (IN738 side), due to the relatively high hardness of the IN738 base metal itself, the hard and brittle phases in this region are mainly Ni-Cr borides, and their impairing effect on performance is relatively controllable; in the DAZ (Hastelloy X side), the nanohardness of the Hastelloy X is low (5.45 GPa), while Ni-Cr borides (P10) and Cr-Mo borides (9.756 GPa) exhibit high nano-hardness, which also become key factors affecting the overall performance of the joint. In summary, the precipitation morphology, distribution density, and hardness characteristics of hard and brittle borides in ASZ and DAZ are the core microfactors determining the mechanical properties of the joint.

**Figure 9:**
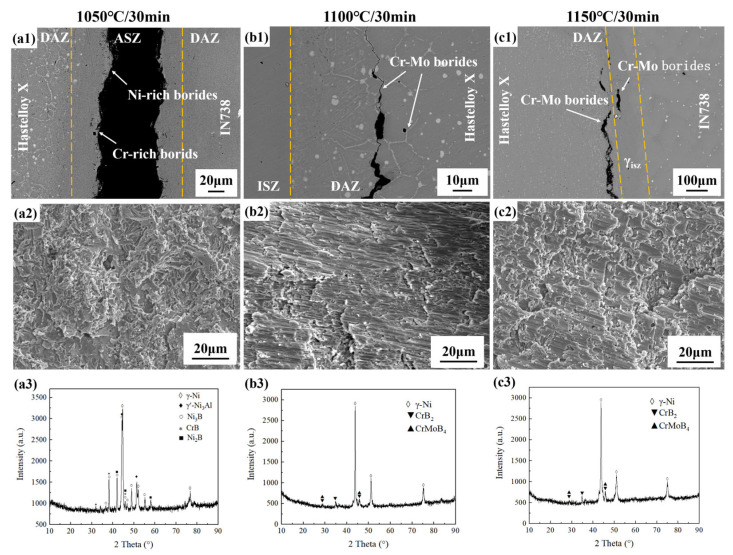
The fracture behaviors of Hastelloy X/IN738 joints bonded at different temperatures for 30 min tested at room temperature (RT): (**a1**–**c1**) cross-sections of joints; (**a2**–**c2**) fracture morphologies of the joints; (**a3**–**c3**) XRD pattern of the joints’ fracture surfaces.

Figure 9 reveals a distinct transition in fracture path with bonding temperature, which is strongly corroborated by the phase identification from XRD analysis (see in Figure 9a3–c3). At 1050 °C, fracture occurred through the ASZ, rich in continuous hard and brittle borides. XRD patterns detected strong diffraction peaks for Cr-rich borides (CrB) and Ni-rich borides (Ni_3_B), confirming that these borides are the primary weak region responsible for the joint’s premature failure. When brazed at 1100 °C or 1150 °C, cracks propagated along Cr-Mo borides in the DAZ (Hastelloy X side), leading to the joints’ fracture. At 1100 °C, complete isothermal solidification yielded a ductile γ-Ni seam; XRD pattern (see in Figure 9b3) showed Cr-rich/Ni-rich boride peaks (marked with circles “○”) vanished, replaced by Cr-Mo boride signals (marked with “▲”). At 1150 °C, XRD confirmed that Cr-Mo borides remained stable and fracture-controlling, while the significantly enhanced γ-Ni diffraction peaks indicated improved microstructural homogeneity.

**Figure 10:**
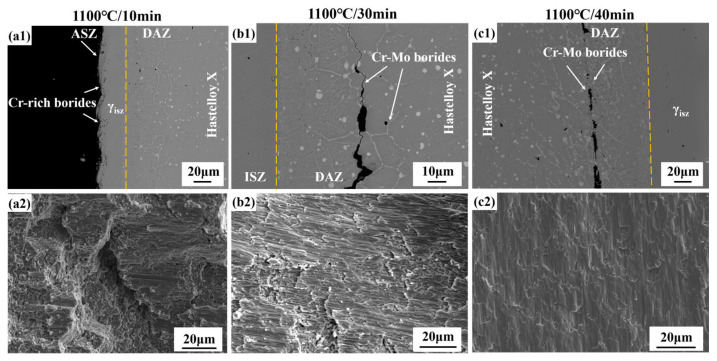
The fracture behaviors of Hastelloy X/IN738 joints bonded at 1100 °C for 10 min, 30 min and 40 min tested at RT: (**a1**–**c1**) Cross sections of joints; (**a2**–**c2**) Fracture morphologies of the joints.

Figure 10 shows typical cross-sections and fracture morphologies of Hastelloy X/IN738 joints (tested at RT) bonded at 1100 °C for different bonding times. When brazed at 1100 °C/10 min, isothermal solidification was not achieved in the joint, and the fracture path propagated along the ASZ. According to nano-indentation test results, the Cr-rich borides in the ASZ exhibit extremely high hardness (15.919 GPa) and poor ductility. It can be inferred that during shearing, high stress concentration occurs at the interfaces between the Cr-rich borides and the γ-Ni matrix, leading to the formation of micro-cracks around the Cr-rich borides. The joint brazed at 1100 °C/10 min failed along continuous Cr-rich borides and exhibited a brittle fracture mode, with few dimples observed in the fracture morphologies (see in Figure 10a1).

When joints were brazed for 30 min and 40 min, complete isothermal solidification joints were achieved (see in Figure 6c1,d1), effectively eliminating the hard and brittle phases in ASZ. Consequently, the fracture path shifted to the DAZ on the side of Hastelloy X, propagating primarily along the Cr-Mo borides. However, distinct differences in fracture behavior were observed between the two conditions, which correlated directly with the enhancement in mechanical properties. When brazed at 30 min, crack propagation exhibited a mixed-mode characteristic, comprising both intergranular and transgranular fracture (see in Figure 10b1). Cracks propagated predominantly along grain boundaries decorated with Cr-Mo borides (intergranular fracture), whereas in localized regions, stress concentration at the boride/matrix interface induced transgranular crack penetration through the grain interiors. This indicates that the interfacial bonding between the Cr-Mo borides and the γ matrix remained relatively weak, and the intrinsic brittleness of the borides dominated the fracture process. The corresponding fracture surface exhibited few and shallow dimples, consistent with a moderate strength level.

Notably, when the bonding time was extended to 40 min, the Cr-Mo borides exhibited significant plastic deformation—a phenomenon not observed in the 30 min-bonding joint. This capability for plastic deformation is a direct consequence of the microstructural evolution described earlier (Section 3.3): the coarsening and spheroidization of Cr-Mo borides, driven by extended interdiffusion (particularly of Mo). As seen in Figure 10c1, this deformation directly indicates improved interfacial compatibility. Thus, the sufficient diffusion of Mo during the extended bonding time facilitated not only morphological changes but also enhanced the intrinsic toughness of the Cr-Mo borides and strengthened their interfacial bonding with the matrix. Research from Zhang, et al. [34] showed that strong Mo-B covalent bonds increased the volume modulus, shear modulus, and reduce Poisson’s ratio, which substantially improved both toughness and plastic deformation capability. Consequently, during fracture propagation along the Cr-Mo boride complexes in the DAZ on the Hastelloy X side, the crack path bends upon encountering the plastically deformed borides rather than propagating linearly. This crack deflection, facilitated by boride plasticity and enhanced interfacial bonding, extends the effective crack path and dissipates additional energy, complementing the increased resistance from boride plastic deformation. The fracture surface exhibits typical ductile characteristics, with larger and deeper dimples, reflecting the combined effects of boride plasticity and crack deflection, which together account for the marked improvement in joint mechanical properties.

In summary, extending the bonding time from 30 to 40 min essentially enhanced the plasticity of the Cr-Mo borides and their compatibility with the matrix by promoting Mo diffusion and compositional homogenization. This transitioned the fracture mechanism from a “brittleness-dominated mixed fracture” to a “ductility-dominated microvoid coalescence fracture,” ultimately leading to the observed strength enhancement.

### 3.5. Boride Precipitation Mechanism in DAZ on the Hastelloy X Side and Microstructural Evolution Model of TLP-Bonding Joint

**Figure 11:**
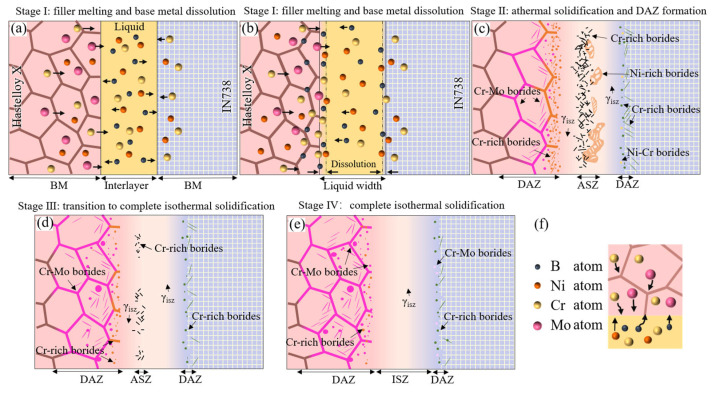
Diagram of the microstructure evolution mechanism of the TLP bonding Hastelloy X/IN738 joint: (**a**) filler melting and element diffusion; (**b**) interface element enrichment; typical microstructure of the brazed joint; (**c**) formation of continuous ASZ; (**d**) formation of discontinuous ASZ; (**e**) formation of complete ISZ; (**f**) Diagram of the element diffusion mode.

The microstructure evolution mechanism of the Hastelloy X/IN738 joint is illustrated in Figure 11.

1. Stage I: filler melting and base metal dissolution (See in Figure 11a,b);

During heating to bonding temperature, the BNi-2 interlayer melted, rapidly wetting and spreading between Hastelloy X and IN738. Subsequently, inter-diffusion of elements occurred between the liquid phase and the base metals (Figure 11b), leading to the dissolution of the base metals.

2. Stage II: athermal solidification and DAZ formation—Exemplified by 1050 °C/30 min (Figure 5a);

At this stage, isothermal solidification initiated, yet insufficient diffusion of B and Si atoms occurred due to the relatively low temperature. Consequently, residual liquid phase rich in MPD elements remained at the joint centerline and solidified during subsequent cooling, forming ASZ in the seam. Simultaneously, B atoms diffused into both base metals and reacted with boride-forming elements (e.g., Cr, Mo), precipitating borides within the diffusion-affected zones (DAZ). On the Hastelloy X side, its abundant grain boundaries provided fast diffusion channels for B atoms. B reacted with the high concentrations of Cr and Mo in the base metal, preferentially precipitating Cr-rich and Cr-Mo borides. This consumption of B at the interface created a B-depleted zone, which further drove the continuous diffusion of B atoms from the liquid phase towards this side. In contrast, on the IN738 side, its dense γ’/γ structure and scarce grain boundaries significantly hindered the diffusion of B atoms. This caused B atoms to accumulate at the interface, thereby reducing the concentration gradient (∇C) and diminishing the diffusion driving force. Ultimately, during cooling, these accumulated B atoms reacted with Ni, forming Ni-rich borides within the ASZ adjacent to the IN738 side. Thus, the microstructure of the ASZ exhibited asymmetry: Cr-rich borides were located near the Hastelloy X side, while Ni-rich borides were situated close to the IN738 side. Concurrently, the DAZ was established: the DAZ (Hastelloy X side) contained blocky and acicular Cr-rich borides, Cr-Mo borides, and a small amount of Ni-Cr borides; whereas DAZ (IN738 side) comprised fine, dot-like and acicular Cr-rich and Ni-Cr borides (see in Figure 11c).

3. Stage III: transition to complete isothermal solidification—Exemplified by 1075 °C/30 min (Figure 5b) or 1100 °C/10 min (Figure 6a1);

As the bonding temperature increased, atomic diffusivity was enhanced. This promoted the isothermal solidification process, reducing the residual liquid phase and resulting in a discontinuous ASZ (see in Figure 11d). The width of the DAZ on both sides increased accordingly. In DAZ (Hastelloy side) the previously formed Cr-rich borides gradually dissolved, while the relatively more stable Cr-Mo borides increased in quantity.

4. Stage IV: complete isothermal solidification (under optimal parameters, e.g., 1100 °C/40 min in Figure 6d1)

With suitable temperature and sufficient holding time, the MPD elements diffused adequately, leading to complete isothermal solidification of the joint. As shown in Figure 11e, the brittle ASZ was completely eliminated. The joint consisted of DAZ (Hastelloy X side), ISZ and DAZ (IN738 side). The joint’s microstructure is: Hastelloy X substrate/(Cr-Mo borides + Cr-rich borides)/γ_ISZ_/(Cr-rich borides + Cr-Mo borides)/IN738 substrate.

It is worth noting that the precipitation location and distribution of borides in the joint follow distinct patterns. In the ASZ region, the distribution of Cr-rich borides and Ni-rich borides shows a significant bias: Cr-rich borides tend to precipitate preferentially near the Hastelloy side. This is closely related to the rapid diffusion of B atoms on the Hastelloy side. B atoms enriches preferentially on this side, causing B in the residual liquid phase to react preferentially with Cr diffused from the Hastelloy side, thereby promoting the formation of Cr-rich borides. In DAZ (Hastelloy X side), the precipitation of Cr-Mo borides and Cr-rich borides also follows specific mechanisms. The precipitation of borides in the DAZ on the Hastelloy X side is governed by the distinct diffusion behaviors of B, Cr, and Mo. Owing to their small atomic size and high diffusivity, B atoms rapidly enter the base metal and reacts preferentially with Cr near the joint interface, forming fine, dot-like Cr-rich borides. In contrast, the larger atomic radius of Mo results in significantly slower bulk diffusion. Mo atoms primarily migrate through grain boundaries and, following long-range diffusion, combine with B and Cr at these high-energy sites to form blocky or continuous Cr–Mo borides, which typically precipitate farther from the joint than the Cr-rich borides.

## 4. Conclusions

1. The microstructure of the Hastelloy X/IN738 joint brazed at 1050 °C for 30 min consists of ISZ, ASZ, and DAZ. The ISZ contains γ_ISZ_, while the ASZ comprises γ, Ni-rich borides, and Cr-rich borides. The borides in DAZ (Hastelloy X side) contain Cr-Mo borides preferentially precipitated along grain boundaries, intragranular dot-like Cr-Mo borides, and a small amount of Cr-rich borides. On the IN738 side, the primary precipitates are Ni-Cr borides.
2. The high density of grain boundaries in Hastelloy X significantly enhances B diffusion, thereby promoting extensive boride formation in DAZ (Hastelloy X side). The advantageous chemistry (high Cr, Mo content) and microstructure (abundant grain boundaries) of Hastelloy X both lead to the unique phenomenon of biased Cr-boride precipitation on this side.
3. With the increase in bonding temperature, enhanced diffusion of elements (e.g., B, Si) improves the completeness of isothermal solidification in the joint, resulting in the dissolution of Cr-borides and Ni-borides in the ASZ; however, excessive temperature causes base metal dissolution, leading to a significant increase in the diffusion distance and the re-precipitation of Ni-rich borides and Cr-rich borides. Prolonging the holding time further improves the degree of isothermal solidification and promotes the sufficient diffusion of Mo elements.
4. The Cr-rich borides and Ni-rich borides in the ASZ, and the Cr-Mo boride regions in the DAZ (Hastelloy X side) exhibit much higher nano-hardness, reaching 10.301, 15.919, and 9.756 GPa, respectively. These borides in the ASZ and DAZ impair the shear strength of the joint.
5. The RT shear strength of the joint first increases and then decreases with the rise in bonding temperature, and increases with the extension of bonding time. With the temperature elevated to 1100 °C, the hard and brittle borides in the ASZ are progressively dissolved, thereby increasing the joint strength to 476 MPa. Excessively high temperature induces the re-precipitation of borides in the seam, resulting in a decrease in joint strength. With the prolongation of bonding time, the Cr-rich borides in the ASZ gradually decrease, and complete isothermal solidification is achieved at 30 min. Further extending the bonding time facilitates the full diffusion of Mo elements, which strengthens the toughness of Cr-Mo borides and their interfacial bonding with the matrix, leading to a significant improvement in joint toughness. The maximum room-temperature shear strength of 587 MPa is obtained when the joint is brazed at 1100 °C for 40 min.

## Figures and Tables

**Table 1:** The elements of raw materials and BNi-2 filler (wt. %).

Alloy	Ni	Co	Fe	Cr	Mo	W	Al	C	B	Si
IN738	Balance	8.4	-	16.0	1.7	2.7	3.4	0.1	-	-
Hastelloy X	Balance	1.8	17.5	21.0	8.5	0.6	0	0.1	-	-
BNi-2	Balance	-	3.0	7.0	-	-	-	-	3.1	4.5

**Table 2:** Elemental compositions (at. %) of various phases marked in Figure 2.

Point	C	Al	Si	Mo	Ti	Cr	Fe	Co	Ni	W	Possible Phase
1	7.13	1.22	1.56	0.44	2.44	5.36	2.32	1.07	77.21	1.24	Ni-rich borides
2	1.68	0.94	0.81	6.38	1.04	80.36	0.29	0.31	6.49	1.70	Cr-rich borides
3	5.09	0.45	6.15	0.29	0.40	9.36	4.33	1.18	71.5	1.26	γ-Ni
4	0.80	0.00	0.00	31.55	1.0	27.27	7.09	1.51	26.6	4.18	Cr-Mo borides
5	1.21	0.00	3.12	36.39	1.02	23.27	7.12	1.14	24.6	2.13	Cr-Mo borides
6	4.25	0.79	1.75	7.99	0.21	32.24	12.04	1.16	38.30	1.27	Ni-Cr borides
7	9.69	0.65	0.24	10.72	0.19	64.14	5.85	0.54	6.85	1.13	Cr-rich borides
8	8.28	2.92	9.67	0.60	1.96	10.52	1.86	3.91	59.23	1.05	Ni-rich borides
9	3.93	1.22	10.15	8.10	1.20	41.74	0.94	2.28	24.58	5.86	Ni-Cr borides

## Data Availability

The original contributions presented in this study are included in the article. Further inquiries can be directed to the corresponding authors.

## Abbreviations

The following abbreviations are used in this manuscript:

ASZ	athermal solidification zone
DAZ	diffusion affected zone
ISZ	isothermal solidification zone
TLP-Bonding	transient liquid phase Bonding
MPD	melting point depressant
SEM	scanning electron microscope
EDS	energy dispersive spectroscopy
EPMA	electron probe microanalyzer
XRD	X-ray diffraction
RT	room temperature

## References

[1] García-Martínez M., del Hoyo Gordillo J.C., Valles González M.P., Pastor Muro A., González Caballero B. (2023). Failure study of an aircraft engine high pressure turbine (HPT) first stage blade. Eng. Fail. Anal..

[2] Yan W., Li T., Xing X., Wang X., Liu D. (2025). Experimental study on surface temperature and emissivity of rotating turbine blades of a micro turbojet engine. Appl. Therm. Eng..

[3] Zhang L., Chen J., Yang S., Lu H., Qiu C., Zhang Y., Zheng Y., Qin R. (2025). Review on high-energy beam repair of surface damages in Ni-based single crystal superalloys. Eng. Fail. Anal..

[4] Kim B.-H., Kong B.-O., Joo Y.-K., Son I.-S., Hong H.-U., Lee J.-H. (2024). The influence of γ′ morphology and size on stress rupture properties in Ni-base superalloy IN738LC. J. Mater. Res. Technol..

[5] Zou T., Lang Z., Lu R., Liu J., Xu X. (2025). Tensile deformation and fracture behavior at high temperature of the TLP joint for Hastelloy X superalloy with laminated interlayer. Eng. Fail. Anal..

[6] Peng J., Wu R., Zhang J., Cai H. (2023). High-temperature mechanical properties and microstructure of welded joint in GH4169/IC10 dissimilar nickel-based superalloys by vacuum electron beam welding. Mater. Sci. Eng. A.

[7] Sun W., Wang S., Xin J., Tan G., Hong M., Wu M., Ke L. (2020). Microstructure and mechanical properties of the IC10/GH3039 dissimilar electron beam welded joint. Vacuum.

[8] Wu T., Zhang Q., Lu H., Shi Y., Zhang Q., Zhang S., Wang R., Lin P., Lin T., He P. (2023). Diffusion brazing of GH536 polycrystalline superalloy with IC10 single crystal superalloy using BNi-2 interlayer. J. Mater. Res. Technol..

[9] Zhang Y., Guo B., Li J., Wang Z., Wang J. (2023). Predicting solidification cracking in directed energy deposition of Hastelloy X alloys based on thermal-mechanical model. J. Manuf. Process..

[10] Ardeshiri A., Razavi S.H., Khodabakhshi M., Ashiri R. (2025). Optimization and insights toward fully dense crack-free additive manufacturing of Hastelloy-X by selective laser melting technology. J. Mater. Res. Technol..

[11] Chen M., Hua L., Hu Z., Dong K., Qin X. (2025). Cracking and suppression mechanisms of directed energy deposited IN738 superalloy revealed by microstructural characterization, in-situ thermal monitoring, and numerical simulations. J. Alloys Compd..

[12] Hou X., Wang S., Qiu K., Sun Y., Yang Y., Zhou Y. (2022). Influence of Post-Bond Heat Treatment on Microstructure and Creep Behavior of the Brazed Single-Crystal Nickel Superalloy. Materials.

[13] Sun Y., Wang Z. (2023). The effect of melting point depressant elements B, Si, and P in Ni-based brazing filler metals on the formation of brazed joints. Weld. World.

[14] Yarmou Shamsabadi A., Farvizi M., Nikzad L., Malekan A. (2025). Dissimilar TLP bonding of X-45/Hastelloy X superalloys using BNi-2 filler metal: Microstructural evolution and mechanical behaviors. J. Adv. Join. Process..

[15] Zhang Y., Cheng Y., Zhong Y., He N., He L., Gao Z., Gong X., Chen C., Ye H. (2024). Precipitation evolution of M5B3 boride in diffusion affected zone and its effect on mechanical properties of TLP bonded Mar-M247 superalloys. Mater. Sci. Eng. A.

[16] Li S., Li J., Shi J., Peng Y., Peng X., Sun X., Jin F., Xiong J., Zhang F. (2022). Microstructure and mechanical properties of transient liquid phase bonding DD5 single-crystal superalloy to CrCoNi-based medium-entropy alloy. J. Mater. Sci. Technol..

[17] Cook G.O., Sorensen C.D. (2011). Overview of transient liquid phase and partial transient liquid phase bonding. J. Mater. Sci..

[18] Chen B., Xiong H.-P., Mao W., Cheng Y.-Y., Wu X. (2015). Dissimilar joining of P/M superalloy and single crystal superalloy using Ni–Cr–B brazing alloy. Weld. World.

[19] Wang G., Sun Y., Wang X., Liu J., Liu J., Li J., Yu J., Zhou Y., Jin T., Sun X. (2017). Microstructure evolution and mechanical behavior of Ni-based single crystal superalloy joint brazed with mixed powder at elevated temperature. J. Mater. Sci. Technol..

[20] Li S., Peng Y., Du Y., Yuan L., Xiong J., Li J. (2022). Microstructural characteristics and mechanical properties of IC10 superalloy and (CoCrNi)94Al3Ti3 MEA joint brazed using NiCrSiB filler. Mater. Charact..

[21] He Q., Zhu D., Dong D., Xu M., Wang A., Sun Q. (2019). Effect of Bonding Temperature on Microstructure and Mechanical Properties during TLP Bonding of GH4169 Superalloy. Appl. Sci..

[22] Sun Z., Chen X., Zhang L., Zhang S., Feng J. (2021). Experimental and Numerical Study of Transient Liquid Phase Diffusion Bonded DZ40M Superalloys. Crystals.

[23] Malekan A., Farvizi M., Mirsalehi S.E., Saito N., Nakashima K. (2019). Influence of bonding time on the transient liquid phase bonding behavior of Hastelloy X using Ni-Cr-B-Si-Fe filler alloy. Mater. Sci. Eng. A.

[24] Lin Y., Jiangtao X., Yajie D., Jin R., Junmiao S., Jinglong L. (2021). Microstructure and mechanical properties in the TLP joint of FeCoNiTiAl and Inconel 718 alloys using BNi2 filler. J. Mater. Sci. Technol..

[25] Nasajpour A., Farzadi A., Mirsalehi S.E. (2022). Effect of diffusion brazing time on microstructure, isothermal solidification completion and microhardness distribution during joining of Nicrofer 5520 superalloy using a liquated Ni–Cr–B interlayer. J. Mater. Res. Technol..

[26] Gogebakan M., Kursun C., Gunduz K.O., Tarakci M., Gencer Y. (2015). Microstructural and mechanical properties of binary Ni–Si eutectic alloys. J. Alloys Compd..

[27] Pouranvari M., Ekrami A., Kokabi A.H. (2013). Solidification and solid state phenomena during TLP bonding of IN718 superalloy using Ni–Si–B ternary filler alloy. J. Alloys Compd..

[28] Sun H., Fu W., Song X., Tian X., Wu G., Chen X., Wang H. (2024). Brazing of high-nitrogen steel to Al_0.3_CoCrFeNi using a BNi-2 filler: Microstructure, mechanical properties, and corrosion resistance. J. Mater. Res. Technol..

[29] Arhami F., Mirsalehi S.E., Sadeghian A. (2019). Effect of bonding time on microstructure and mechanical properties of diffusion brazed IN-939. J. Mater. Process. Technol..

[30] Xu R., Wu T., Li X., Zhang X., Cui J., Lu F., Zhang S., Wang C., Lin P., He P. (2024). High-strength stainless steel joints achieved by co-regulation mechanism of phosphide dispersed distribution and γ-phase generation. Mater. Charact..

[31] Li X.C., Sun J., Liu Y.Z., Fu W., Song X.G., Yang S.R., Long F., Hu S.P. (2025). Microstructure and mechanical properties of CoCrNi/GH99 medium entropy alloy brazed joints: Formation of medium entropy brazing seam. Intermetallics.

[32] Chen J., Hu M., Li C., Li H., Xia X. (2025). Effect of Post Weld Heat Treatment on Microstructure and Creep Property of the TLP Bonded Ni-Based Superalloy. Met. Mater. Int..

[33] Zhang Z.P., Liu J.D., Qiu K.Q., Huang Y.Y., Li J.G., Wang X.G., Liu J.L., Wang M., Zou M.K., Zhou Y.Z. (2023). Effects of Brazing Temperature on Microstructure and High-Temperature Strength of Joints Using a Novel Fourth-Generation Nickel-Based Single Crystal Superalloy. Met. Mater. Int..

[34] Zhang M., Wang H., Wang H., Cui T., Ma Y. (2010). Structural Modifications and Mechanical Properties of Molybdenum Borides from First Principles. J. Phys. Chem. C.

